# The Royal Institute of Public Health

**Published:** 1920-01-24

**Authors:** 


### The Royal Institute of Public Health.

A SPECIAL COURSE OF LECTURES AND DEMONSTRATIONS ON TUBERCULOSIS.

Lecture I., Thursday, January 15, 5 p.m.: "Schemes and Methods in Tuberculosis Work," by Professor E. W. Hope, O.B.E., M.D., D.Sc.

Lecture II., January 22: "A Practical Demonstration of the Work of a Tuberculosis Department at Poplar" (at Poplar Dispensary for the Prevention of Consumption, 135 Bow Road, E. 3), by T. D. Lister, Esq., M.D., M.R.C.P., etc.

Lecture III., January 29: "Tuberculosis in Relation to Life Assurance," by Otto May, Esq., M.D., M.R.C.P.

Lecture IV., February 5: "Climate in Tuberculosis," by E. C. Morland, Esq., M.B., B.Sc. Lond., M.D. Berne.

Lecture V., February 12: "The Work of a Tuberculosis Department," by Donald P. Sutherland, Esq., M.B., B.S.

Lecture VI., February 19: "The Problem of Immunity in Tuberculosis," by S. Roodhouse Gloyne, Esq., M.D., D.P.H.

Lecture VII., February 26: "Some Pathological Aspects of Tuberculosis," by W. E. Carnegie Dickson, Esq., M.D., B.Sc., F.R.C.P.

Lecture VIII., March 4: "Hospital Treatment of Pulmonary Tuberculosis," by T. Gwyne Maitland, Esq., M.A., M.D.

Lecture IX., March 11: "X-ray in the Diagnosis of Tuberculosis," by H. Martin Berry, Esq., M.D.

Lecture X., March 18: "Suggested Reforms in the Campaign against Tuberculosis," by S. Vere Pearson, Esq., M.D., M.R.C.P.

Bacteriological demonstrations will be given in the laboratories of the Institute, and visits will be arranged to sanatoria, tuberculosis dispensaries, etc. The lectures will be delivered in the Lecture Room of the Institute on Thursdays at 5 p.m. An optional examination will be held at the close of the course, at which certificates will be awarded to successful candidates. Fee for the course, two guineas. Fellows and members admitted free. Further information may be obtained on application.

E. W. Hope, M.D., D.Sc.,
T. N. Kelynack, M.D., M.R.C.P.,
Hon. Secretaries.

